# Compressible, Flame-Resistant and Thermally Insulating Fiber-Reinforced Polybenzoxazine Aerogel Composites

**DOI:** 10.3390/ma13122809

**Published:** 2020-06-22

**Authors:** Yunyun Xiao, Liangjun Li, Fengqi Liu, Sizhao Zhang, Junzong Feng, Yonggang Jiang, Jian Feng

**Affiliations:** 1 Science and Technology on Advanced Ceramic Fibers and Composites Laboratory, College of Aerospace Science and Technology, National University of Defense Technology, Changsha 410073, China; xyy8407@126.com (Y.X.); liufengqi13@nudt.edu.cn (F.L.); junzongfeng@nudt.edu.cn (J.F.); jygemail@163.com (Y.J.); 2 China-Australia International Institute for Mineral, Metallurgy and Materials, Jiangxi University of Science and Technology, Nanchang 330013, China; zhangsz@jxust.edu.cn

**Keywords:** polybenzoxazine aerogels, fiber reinforcement, thermal insulation, flame-retardancy

## Abstract

The preparation of novel polymer aerogel materials with enhanced flame-retardancy, superior thermal insulation and mechanical strength is of great practical significance in energy-savings and fire-prevention for buildings. Herein, we reported the fiber-reinforced polybenzoxazine (PBO) aerogel composites with flame retardance and thermal insulation, which were prepared under room temperature and atmospheric pressure, and using 4,4′-diaminodiphenlymethane (MDA) benzoxazine monomer as the raw material and oxalic acid (OA) as the catalyst. Several outstanding attributes were achieved in the aerogel composites, such as relatively low thermal conductivity (0.069 W/m·K at 10^5^ Pa, 0.031 W/m·K at 5 Pa), high limiting oxygen index (LOI) up to 32.5, and enhanced mechanical properties. It can be compressed to more than 80% of the deformation without obvious cracks, and shows high compressive modulus and specific modulus (20.69 MPa and 5.05 × 10^4^ N·m/Kg, respectively). All the excellent comprehensive properties mean that fiber-reinforced PBO aerogel composites have broad application prospects in the fields of flame retardancy and thermal insulation.

## 1. Introduction

Aerogels have been widely used in civil, residential and industrial buildings, due to their low density, and excellent thermal insulation and acoustic insulation [1,2,3]. Inorganic aerogels, such as SiO_2_ and Al_2_O_3_ aerogels, possess superior thermal insulation. They are also noninflammable. However, they are disadvantaged by poor mechanical properties and easy dust release, requiring enhancement by fiber or some other enhancer in order to implement their application [4,5,6,7]. However, their intrinsic fragility is challenging to overcome [8,9]. Organic polymer aerogels are more advantageous because of their good formability, low thermal conductivity and excellent mechanical properties [10,11]. Moreover, the flexibility of polymers can provide comfort to furniture in houses and seats in vehicles. Unfortunately, their innate organic nature, low density and high porosity put them at risk of fire, because they can be ignited easily and burn at a high speed, thus severely affecting the safety of people’s lives and property. Therefore, improving the flame-retardancy of polymer aerogels has important practical significance.

Generally, the flame-retardancy of polymer aerogels can be improved effectively by adding inorganic flame-retardant ingredients (clay [12,13], graphene oxide [14], montmorillonite [15,16], etc.) or grafting a flame-retardant chain [17] in the polymers. These methods can effectively improve polymeric flame-retardancy. However, the mechanical strength of polymer aerogels is weakened due to the poor compatibility between inorganic components and the polymers matrix. Hence, it is still a challenge to prepare flame-retardant and thermally insulating polymer aerogels with superior mechanical properties.

On the other hand, designing and fabricating polymer aerogels with inherent flame-retardancy, while maintaining the self-extinguishing and excellent overall properties of polymer aerogels, is also a meaningful way to improve the flame-resistance of polymers [17]. Polybenzoxazine (PBO) aerogel is a new type of phenolic polymer aerogel material, which possesses intrinsic flame-resistance due to abundant benzene rings and nitrogen atoms in the structure [18,19,20]. The intrinsic flame-resistance of PBO, combined with the thermal insulation of aerogel materials, makes PBO aerogel a promising material for flame-retardant and thermal insulation [21,22]. Recently, we reported on PBO aerogels derived from a 4,4′-diaminodiphenlymethane (MDA) monomer, prepared by supercritical drying and using HCl as the catalyst under room temperature, and systematically studied their structure, thermal insulation and mechanical properties [23]. In this work, HCl is highly corrosive, volatile, and is harmful to humans and the environment, and the supercritical drying is a safety risking. Thus, using oxalic acid (OA) (as a catalyst) and atmospheric pressure drying to prepare PBO aerogels is of great significance. Moreover, the flame-retardant properties of PBO aerogels have not been studied, to our best knowledge. The char yield is a critical factor in evaluating the flame-retardant properties of materials, and a high char yield indicates that the materials have excellent flame-retardant properties. However, the char yield of PBO aerogels based on an MDA monomer was about 40% [23], and that of PBO resin based on the same monomer was only 48% at 800 °C [24]. Thus, the flame-retardancy of PBO aerogels needs to be further improved. Furthermore, there are few reports on the use of fiber reinforcement to improve the strength and flame-retardancy of PBO aerogels, or to overcome the mismatch of inorganic components, such as particles and whiskers, with the PBO aerogel matrix.

In this paper, mullite fiber was used as the reinforced phase, by vacuum-impregnation of PBO sol, to prepare fiber-reinforced PBO aerogel composites through gelling aging, solvent-exchanging and ambient pressure drying. Mullite fiber is an excellent reinforcer, because of its light weight, high temperature resistance and noninflammability. After compounding with PBO aerogels that are self-extinguishing and have low thermal conductivity, the composite aerogels can ensure low density and excellent mechanical properties, while further improving flame-retardancy.

## 2. Experimental Section

4,4′-diaminodiphenlymethane (MDA) benzoxazine monomers (solid, CP) were purchased from Coryes Polymer Sci. & Tech Co., Ltd. (Chengdu, China). Mullite fibers (The real density is about 3.4 g/cm^3^) were obtained from Shandong Luyang energy-saving materials Co., Ltd. (Zibo, China). N, N-dimethylformamide (DMF, AR), ethanol (AR), n-hexane (AR) and OA dehydrate (AR) were purchased from Sinopharm Chemical Reagent Co., Ltd. (Shanghai, China). All the reagents were used without further purification.

MDA benzoxazine monomer was added to the DMF solvent and mixed via magnetic stirring until completely dissolved. Afterward, the OA (7.15 mol/L in DMF) was added to this solution and stirred for 20 min to form PBO sol. The molar ratio of MDA:OA:DMF was 1:3.76:27.30. Subsequently, mullite fiber, with the size of 100 × 100 × 20 mm^3^ and the density of 0.18g/cm^3^ (volume fraction is 5%), was impregnated to PBO sol under vacuum (about 100 Pa), and then the prepared mold was sealed under environmental conditions. The gelation time was approximately 50 h, and the composite gels were aged for 96 h. After aging, the solvent was replaced with fresh ethanol, then non-polar *n*-hexane three times (each 12 h). Finally, the aerogel composites were obtained by ambient pressure drying at room temperature, until the weight remained constant and optimized by heating at 80 °C for 24 h. 

The density of the fiber-reinforced PBO aerogel composites was calculated based on the measured volume and weight. The microstructures were observed by field emission scanning electron microscopy (FESEM, S4800, Hitachi, Tokyo, Japan). Compression stress-strain curves were obtained by compressing 20 × 20 × 20-mm^3^ samples at a rate of 0.5 mm/min. The thermal conductivity at 5 and 10^5^ Pa under different temperatures (22, 50, 75 and 100 °C) was measured for triplicate tests using a Hot Disk TPS2500 (5501 Sensor, R = 6.403 mm, Sweden hot disk Co., Ltd, Uppsala, Sweden) thermal constant analyzer equipped with a self-made pressure and temperature-control device. The limit oxygen value was tested according to GB/T2406.2-2009 (Oxygen index method for plastic to determine combustion behavior), a Chinese standard with international relationship to ISO 4589-2 (1996-07) [25].

## 3. Results and Discussion

**Figure 1:**
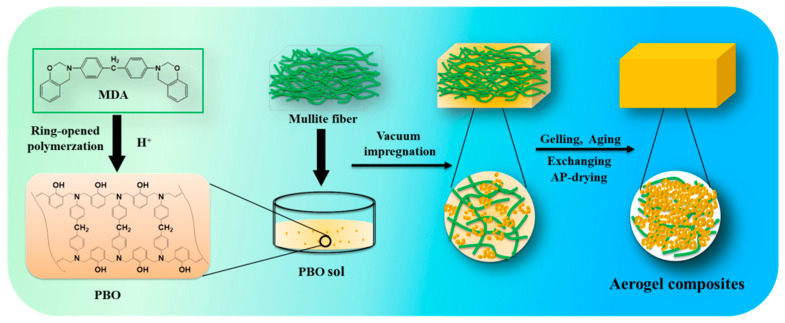
Illustration of the synthesis and structural composition of fiber-reinforced PBO aerogel composites.

**Figure 2:**
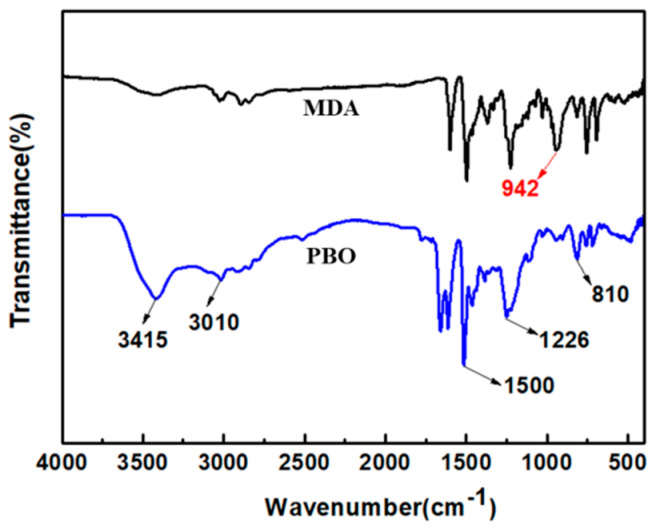
FT-IR spectra of MDA monomer and PBO aerogels.

Figure 1 shows the schematic illustration of the synthesis and structural composition of fiber-reinforced PBO aerogel composites. Firstly, the MDA benzoxazine monomer was subjected to catalytic ring-opening polymerization to form PBO sol, by H^+^ derived from OA. Secondly, as the polymerization continued, the PBO sol particles grew to produce a gel with a three-dimensional network structure. Thirdly, the gel filled the pores among the fibers, and adhered to the surface of the fibers to obtain stable aerogel composites. The FT-IR spectra of the MDA monomer and PBO aerogel are shown in Figure 2; all of the characteristic absorption peaks are basically consistent with those of PBO aerogels reported in our previous study [23], wherein the peaks at 3415, 3010 and 1226 cm^−1^ are attributed to the symmetric stretching of O–H, anti-symmetric stretching vibrations for C–H [22,26], and Ph−O−C stretches [27], respectively, in both MDA monomer and PBO aerogels. The 1500–1700 cm^−1^ region is dominated by C=C (benzene ring), and is difficult to interpret. It is remarkable that the relatively strong absorption peak at 942 cm^−1^ belongs to the stretches of the cyclic acetal in MDA, which disappear in PBO aerogels, indicating that the MDA has been polymerized via ring-opening to form PBO [28].

**Figure 3:**
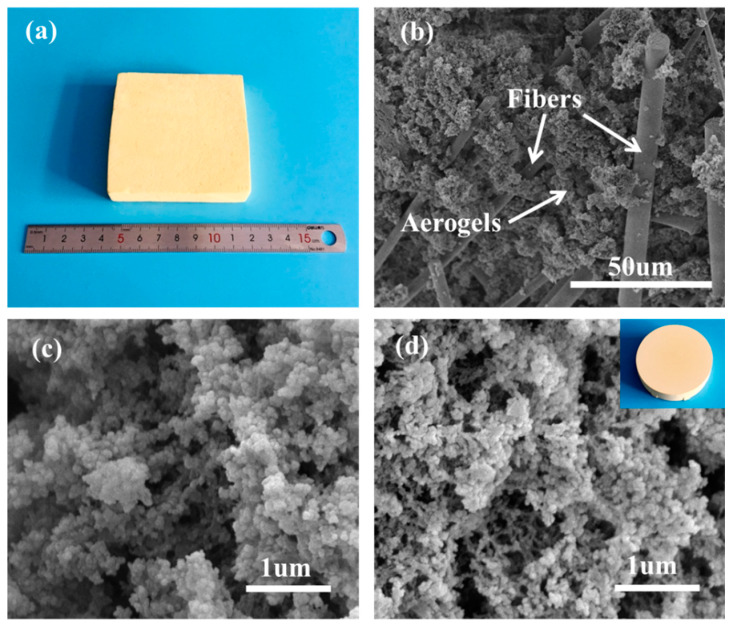
(**a**) photograph of composites, (**b**) SEM image of composites, (**c**) SEM image of PBO aerogel matrix in composites, (**d**) photograph and SEM image of pure PBO aerogel.

The photographs and SEM images of PBO aerogel and fiber-reinforced PBO aerogel composites are shown in Figure 3. Both the aerogel and composite appear straw-yellow, and their densities are 0.29 and 0.41 g/cm^3^, respectively. As shown in Figure 3a, there are no apparent defects or aerogel shedding in the composites. As can be seen from Figure 3b–d, the composites consist of the PBO aerogel’s matrix and mullite fibers, presenting excellent compatibility between them, as expected. The aerogel matrix exhibits a similar 3D nano-porous network structure to that of the PBO aerogel. The mullite fibers are randomly distributed in the PBO aerogel matrix as the backbone of the composite, and the aerogel is well bonded with reinforced fiber.

**Figure 4:**
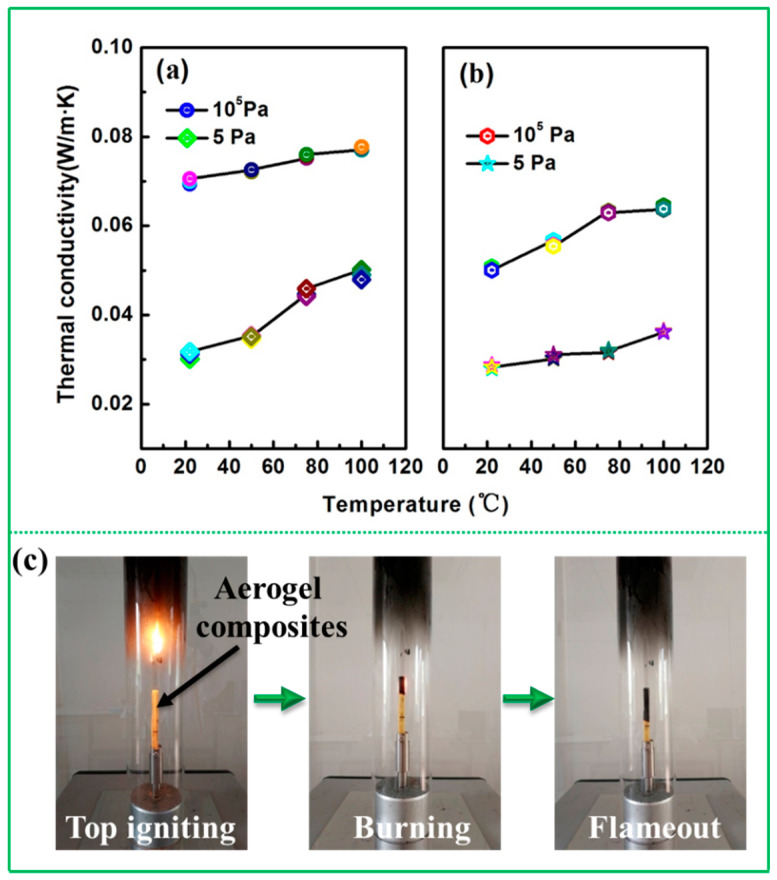
Thermal conductivities of the composites (**a**) and PBO aerogel (**b**) at 5 and 10^5^ Pa under the different temperatures of 22, 50, 75 and 100 °C (**c**). Photos of PBO aerogel composites during the LOI test.

The thermal conductivities of the composites and PBO aerogel, at 5 and 10^5^ Pa and under different temperatures (22, 50, 75 and 100 °C), are depicted in Figure 4. The thermal conductivity of the two samples shows the same trend with a variety of pressures and temperatures. Furthermore, the thermal conductivity of the composites is slightly higher than that of the PBO aerogel under the same environmental conditions. This may be attributed to different densities and structures between PBO aerogels and composites. Generally, the thermal conductivity of aerogel materials is mainly provided by solid, gaseous and radiative heat transfers. When the pressure is 5 Pa, there are few gas molecules (which is close to a vacuum state), so the heat transfer is mainly composed of solid heat transfer and radiant heat transfer that is affected by temperature greatly. It can be observed from Figure 4a,b that as the temperature rose, the thermal conductivity of both the composite and aerogel showed an increasing trend. However, the increase is slight, and the thermal conductivity of the composite is just 0.077 W/m·K at 100 °C under 10^5^ Pa (0.069 W/m·K, 22 °C), exhibiting a relatively good thermal insulation performance. PBO aerogels based on the MDA monomer, with the lower thermal conductivity of 0.035–0.057 W/m·K, were investigated in our previous study [20]. Indeed, low thermal conductivity can be obtained through adjusting the density. However, the mechanical strength decreased rapidly, and the char yield dropped slightly as the density decreased. Thus, the desirable densities of PBO aerogels were used as the matrix for the composites in this study to ensure superior mechanical and flame-retardant properties. The detailed data of thermal conductivities for composites and PBO aerogels are listed in Table 1.

The limit oxygen index (LOI) was used to measure the flame-retardancy of the composite. The test photos are shown in Figure 4c. The LOI of the composite is 32.5, obviously higher than that of the inorganic modified Phenolic- [29], polypropylene- [30] and polyvinyl alcohol-based aerogel composites [31,32], indicating that fiber-reinforced PBO aerogel composites possess superior flame-retardancy.

**Figure 5:**
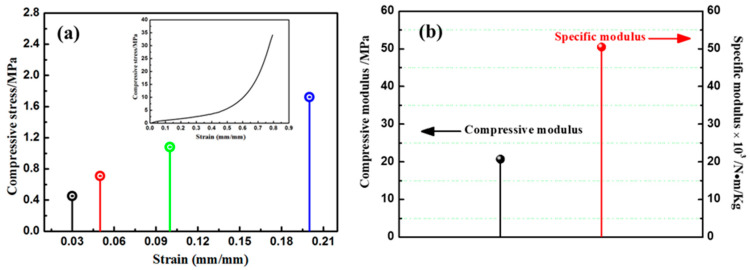
(**a**) Compressive strength at 3%, 5%, 10% and 20% strain, and corresponding compressive stress-strain curve (inset), (**b**) compressive modulus and specific modulus of the composites.

Figure 5 shows the compressive behavior of the PBO aerogel composites. The compressive strength of the composites at 3%, 5%, 10% and 20% strain are 0.45, 0.71, 1.08 and 1.72 MPa, respectively. The inset in Figure 4a presents the compressive stress-strain curve of the composites. With the increase of strain, the stress initially increases slowly, and subsequently rises rapidly without brittle rupture, and its deformation can be up to 80%. The compression curve of the composite material and the fiber-reinforced phenolic aerogel composite material showed the same trend. However, the strength is higher than that of phenolic aerogel composites. The compressive strength of phenolic aerogel composites with a similar density at 3%, 5%, 10% and 20% strain are about 0.25, 0.30, 0.32 and 0.35 MPa, respectively [33] The compressive modulus and specific modulus are 20.69 MPa and 5.05 × 10^4^ N·m/Kg, respectively, as shown in Figure 5b. The composites reveal superior compressive strength due to the excellent integration of the fibers and PBO aerogel matrix.

## 4. Conclusions

In summary, compressible, flame-resistant and thermally insulating fiber-reinforced PBO aerogel composites were prepared, using mullite fiber as the reinforcing backbone and PBO aerogels as the matrix, by vacuum impregnating, sol-gelling and ambient pressure drying. The obtained PBO aerogel composite exhibited an excellent compression performance, with a compressive strength of 1.08 MPa at 10% deformation, relatively low thermal conductivity (0.069 W/m·K, 10^5^ Pa, 22 °C) and superior flame-retardancy, with a LOI of 32.5. The insulation performance of the aerogel composites under different temperatures was also discussed in detail. The thermal conductivity increased slightly with the temperature, and was only 0.077 W/m·K at 100 °C. These excellent properties of the fiber-reinforced PBO aerogel composites will open up the applied prospects for PBO aerogel materials in the field of flame-retardancy and thermal insulation.

## Figures and Tables

**Table 1:** Thermal conductivities for composites and PBO aerogels.

Samples		Average Thermal Conductivities (W/m·K)			
		22 °C	50 °C	75 °C	100 °C
Composites	5 Pa	0.031	0.035	0.044	0.049
	10^5^ Pa	0.069	0.072	0.075	0.077
PBO aerogels	5 Pa	0.028	0.030	0.032	0.036
	10^5^ Pa	0.050	0.055	0.063	0.064
